# Prime-boost-type PspA3 + 2 mucosal vaccine protects cynomolgus macaques from intratracheal challenge with pneumococci

**DOI:** 10.1186/s41232-023-00305-2

**Published:** 2023-11-15

**Authors:** Chieko Yokota, Kosuke Fujimoto, Natsuko Yamakawa, Masamitsu Kono, Daichi Miyaoka, Masaki Shimohigoshi, Miho Uematsu, Miki Watanabe, Yukari Kamei, Akira Sugimoto, Natsuko Kawasaki, Takato Yabuno, Tomotaka Okamura, Eisuke Kuroda, Shigeto Hamaguchi, Shintaro Sato, Muneki Hotomi, Yukihiro Akeda, Ken J. Ishii, Yasuhiro Yasutomi, Kishiko Sunami, Satoshi Uematsu

**Affiliations:** 1 Department of Immunology and Genomics, Graduate School of Medicine, Osaka Metropolitan University, Osaka, Japan; 2 Department of Otolaryngology-Head and Neck Surgery, Graduate School of Medicine, Osaka Metropolitan University, Osaka, Japan; 3 Division of Metagenome Medicine, Human Genome Center, The Institute of Medical Science, The University of Tokyo, Tokyo, Japan; 4 Laboratory of Immunoregulation and Vaccine Research, Tsukuba Primate Research Center, National Institutes of Biomedical Innovation, Health and Nutrition, Ibaraki, Japan; 5 Department of Otorhinolaryngology-Head and Neck Surgery, Wakayama Medical University, Wakayama, Japan; 6 Department of Infection Control and Prevention, Graduate School of Medicine, Osaka University, Osaka, Japan; 7 Division of Fostering Required Medica Human Resources, Center for Infectious Diseases Education and Research (CiDER), Osaka University, Osaka, Japan; 8 Department of Microbiology and Immunology, School of Pharmaceutical Sciences, Wakayama Medical University, Wakayama, Japan; 9 Department of Bacteriology I, National Institute of Infectious Diseases, Tokyo, Japan; 10 Division of Vaccine Science, Department of Microbiology and Immunology, The Institute of Medical Science, The University of Tokyo, Tokyo, Japan; 11 Research Institute for Drug Discovery Science, Osaka Metropolitan University, Osaka, Japan; 12 International Research Center for Infectious Diseases, Osaka Metropolitan University, Osaka, Japan

**Keywords:** *Streptococcus pneumoniae*, Mucosal vaccine, Pneumococcal surface protein A, IgA

## Abstract

**Background:**

Although vaccination is recommended for protection against invasive pneumococcal disease, the frequency of pneumococcal pneumonia is still high worldwide. In fact, no vaccines are effective for all pneumococcal serotypes. Fusion pneumococcal surface protein A (PspA) has been shown to induce a broad range of cross-reactivity with clinical isolates and afford cross-protection against pneumococcal challenge in mice. Furthermore, we developed prime-boost-type mucosal vaccines that induce both antigen-specific IgG in serum and antigen-specific IgA in targeted mucosal organs in previous studies. We investigated whether our prime-boost-type immunization with a fusion PspA was effective against pneumococcal infection in mice and cynomolgus macaques.

**Methods:**

C57BL/6 mice were intramuscularly injected with fusion PspA combined with CpG oligodeoxynucleotides and/or curdlan. Six weeks later, PspA was administered intranasally. Blood and bronchoalveolar lavage fluid were collected and antigen-specific IgG and IgA titers were measured. Some mice were given intranasal *Streptococcus pneumoniae* and the severity of infection was analyzed. Macaques were intramuscularly injected with fusion PspA combined with CpG oligodeoxynucleotides and/or curdlan at week 0 and week 4. Then, 13 or 41 weeks later, PspA was administered intratracheally. Blood and bronchoalveolar lavage fluid were collected and antigen-specific IgG and IgA titers were measured. Some macaques were intranasally administered *S. pneumoniae* and analyzed for the severity of pneumonia.

**Results:**

Serum samples from mice and macaques injected with antigens in combination with CpG oligodeoxynucleotides and/or curdlan contained antigen-specific IgG. Bronchial samples contained antigen-specific IgA after the fusion PspA boosting. This immunization regimen effectively prevented *S. pneumoniae* infection.

**Conclusions:**

Prime-boost-type immunization with a fusion PspA prevented *S. pneumoniae* infection in mice and macaques.

**Supplementary Information:**

The online version contains supplementary material available at 10.1186/s41232-023-00305-2.

## Background

The severe acute respiratory syndrome coronavirus 2 pandemic has significantly increased interest in emerging and re-emerging infectious diseases. With the development of vaccine technology, expectations for effective infectious disease vaccines are increasing.

We previously established intramuscular antigen injection adjuvanted with curdlan + CpG-ODN (prime) and subsequent antigen administration (boost) at target mucosal sites as a new immunization strategy capable of inducing strong and durable systemic and mucosal immunity. This prime-boost vaccine method has been patented in Japan, the USA, and the European Union (patent number: WO2016-199904). We reported that the combination of curdlan + CpG-ODN emulsified with incomplete Freund’s adjuvant (IFA), injected intramuscularly, strongly induced antigen-specific fecal IgA as well as serum IgG and splenic Th1 and Th17 responses [1–3]. In addition, both antigen-specific IgG^+^ and IgA^+^ B cells translocated to lymph nodes throughout the body following immunization with curdlan + CpG-ODN [1]. Even transient antigen-specific IgA production after priming was not detected in respiratory mucosal sites; once primed, high levels of long-lasting antigen-specific lung IgA were induced after intranasal antigen administration [1, 2]. Thus, our prime-boost-type mucosal immunization could be a highly versatile strategy for various respiratory infectious diseases.

Lower respiratory infections accounted for 2.7 million deaths worldwide in 2015, being the third leading cause of years of life lost [4]. Pneumococcal pneumonia was the major cause of death due to lower respiratory infections at all ages [4]. *Streptococcus pneumoniae* is an anaerobic gram-positive bacterium that is the leading cause of pneumonia [5]. The incidence of its infection is highest in children younger than 2 years and adults older than 65 years, and mortality is highest in the elderly [4, 6]. Two types of injectable vaccines are recommended and available for protection against invasive pneumococcal disease: a pneumococcal polysaccharide-protein conjugate vaccine (PCV13) with the 13 most common pediatric capsular serotypes for children and a 23-valent polysaccharide vaccine for adults (PPSV23). Widespread pneumococcal vaccination of children has reduced the overall incidence of invasive disease and hospitalization for pneumonia in all age groups [4, 7]. However, previous studies reported that the widespread use of pneumococcal vaccines increases the infection rates with nonvaccine serotypes [6, 8–10]. There are over 90 different pneumococcal capsular serotypes, and no vaccines are effective for all of them. There is thus a need to develop a universal vaccine that is functional irrespective of the serotype.

Pneumococcal surface protein A (PspA) could represent a promising candidate for the development of a vaccine against *Streptococcus pneumoniae* infection [11]. PspA is one of the most abundant surface molecules of *S. pneumoniae* and prevents phagocytosis by inhibiting complement-mediated opsonization. PspA is categorized into three families and six clades, of which clades 1 and 2 are included in family 1, clades 3–5 in family 2, and clade 6 in family 3. Most of the *S. pneumoniae* isolated from patients belong to families 1 and 2 [12]. Antibodies against PspA exhibit cross-reactivity, but the degree of cross-reactivity varies from clade to clade [5]. Recently, immunization of mice with fusion protein PspA3 + 2 was shown to be induced by antiserum exhibiting a high binding capacity to the clinical isolates and effectively protecting against pneumococcal challenge [12, 13]. Because PspA3 + 2 induced a broad range of cross-reactivity with clinically isolated *S. pneumoniae*, it was thought to be suitable as an antigen for a prime-boost-type mucosal vaccine against pneumococci.

Here, we demonstrate the efficacy of prime-boost-type mucosal vaccine against *S. pneumoniae* in mice and macaques using water-in-oil-in-water (WOW)-emulsified PspA3 + 2 as vaccine antigens. Intramuscular injection of antigen combined with CpG-ODN and curdlan followed by intranasal boosting protected against *S. pneumoniae* infection. Furthermore, a vaccine against *S. pneumoniae* adjuvanted with CpG-ODN and curdlan or curdlan alone induced the production of antigen-specific respiratory SIgA in macaques after intratracheal antigen administration and this vaccine suppressed *S. pneumoniae*-induced pneumonia. Thus, our prime-boost-type mucosal vaccine could be a new preventive strategy against pneumococcal infections.

## Materials and methods

### Animals

Specific pathogen-free female C57BL/6 mice and 2- or 3-year-old macaques were used for this study. Mice were housed in a temperature-controlled (23 ℃ ± 2 ℃) room with a dark period from 8:00 p.m. to 8:00 a.m. They were allowed free access to sterile water and standard laboratory mouse chow. All macaques were kept in the animal facility of Tsukuba Primate Research Center for Medical Science at the National Institute of Biomedical Innovation, Health and Nutrition (NIBIOHN). All animal experiments were performed with the approval of the Osaka Metropolitan University Animal Care and Use Committee and the Animal Committee of NIBIOHN.

### Water-in-oil-in-water emulsion

The composition of WOW was as follows: 36.5% (w/v) squalane (Sigma-Aldrich), 3.5% (w/v) sorbitan sesquioleate (Nikko Chemicals), 0.1% (w/v) sorbitan monooleate (Sigma-Aldrich), and 2.5% (w/v) glycerin (Wako Pure Chemicals Industries) in sterile water.

### PspA3 + 2 expression and purification

Construction of PspA3 + 2 fragments was described previously [12]. Competent *E. coli* BL21 (DE3) cells (TaKaRa) were transformed with pET28a ( +) vectors containing the PspA3 + 2 constructs and were grown in LB medium containing 50 μg/ml kanamycin at 37 °C until the OD_600_ reached 0.7–0.8. Isopropyl β-D-thiogalactoside (IPTG) (Nacalai Tesque) was added until the final concentration reached 0.5 mM, and the culture medium was incubated at 37 °C for 3 h at 180 rpm. After centrifugation at 12,000 × *g* for 15 min, the pellet was washed with sterile deionized water. After another session of centrifugation at 12,000 × *g* for 15 min, the precipitate was resuspended in Buffer A containing 10 mM imidazole (Nacalai Tesque) in 0.1 M PBS (pH 8.0) (Wako) and a protease inhibitor mixture (Roche). The lysate was disrupted by sonication (60 s pulse, 30 s rest, continued for 30 min). After centrifugation at 12,000 × *g* for 10 min, the supernatant was filtered through 0.45 μm filters (Millipore). The supernatants were injected into an ÄKTA Pure 25-equipped HisTrap HP 5 mL column (Cytiva), which was pre-equilibrated with Buffer A. The target proteins were eluted by a gradient of Buffer B containing 500 mM imidazole in 0.1 M PBS (pH 8.0) and a protease inhibitor mixture. The target proteins were collected and desalted using Amicon Ultra-15 10 K (Millipore). The supernatants were filtered through 0.22 μm filters (Millipore) and purified using a HiLoad 26/600 Superdex 200 pg column (Cytiva). The concentration of the target protein was measured using Protein Assay CBB Solution (Nacalai Tesque).

### Intramuscular PspA3 + 2 priming and mucosal boosting regimen in mice

PspA3 + 2 was dissolved alone or in combination with CpG-ODN 1668 (200 μg/mL; Hokkaido System Science) and/or curdlan (20 mg/mL; Invivogen) in phosphate-buffered saline (PBS) and then emulsified in an equivalent volume of WOW. Next, 50 μL of the PBS-WOW emulsion was injected into each of the left and right bicep femoris muscles. Thereafter, blood samples were collected every 2 weeks and subjected to enzyme-linked immunosorbent assay (ELISA) to measure PspA-specific IgG titers, as described below.

Six weeks after the primary immunizations, the mice received PspA boosters administered intranasally. To boost respiratory mucosal immune responses, PBS (5 μL) containing 5 μg of PspA3 + 2 was instilled into each nostril of the mice. Two weeks later, serum and bronchoalveolar lavage fluid (BALF) (1 mL) samples were collected and subjected to ELISA to measure PspA-specific IgG and IgA titers.

### Intramuscular PspA3 + 2 priming and mucosal boosting regimen in macaques

PspA3 + 2 was dissolved alone or in combination with K3-CpG (K3) (200 μg/mL; GeneDesign) and/or curdlan (20 mg/mL) in PBS and then emulsified in an equivalent volume of WOW. Next, 500 μL of the PBS-WOW emulsion was injected into the left deltoid on week 0 and week 4. Blood samples were collected and subjected to ELISA to measure PspA-specific IgG titers, as described below.

At 41 weeks (related to Fig. 3) or 17 weeks (related to Figs. 4 and 5) after the primary immunizations, the macaques received PspA boosters administered intratracheally. To boost respiratory mucosal immune responses, PBS (1 mL) containing 500 μg of PspA3 + 2 was administered intratracheally. After the mucosal boosting immunizations, serum and bronchoalveolar lavage fluid samples were collected and subjected to ELISA for PspA-specific IgG and IgA titer measurements.

### Enzyme-linked immunosorbent assay antibody measurements

Antigen-specific antibody titers in sera and bronchoalveolar lavage fluid were assessed by ELISA. Briefly, 96-well microtiter plates (Nunc-Immuno MicroWell 96-well plates; Thermo Scientific) were coated overnight at 4 ℃ with 1 μg/mL PspA3 + 2 in 100 mM bicarbonate buffer (pH 9.4). The plates were blocked with Block Ace (DS Pharma Biomedical) at room temperature (RT) for 2 h. Test samples serially diluted in Block Ace were added to wells and incubated at RT for 1 h. After washing, horseradish peroxidase-conjugated goat anti-mouse IgG (1000-fold diluted; Southern Biotech), horseradish peroxidase-conjugated goat anti-mouse IgA (1000-fold diluted; Southern Biotech), horseradish peroxidase-conjugated goat anti-monkey IgG (1000-fold diluted; Southern Biotech), and horseradish peroxidase-conjugated goat anti-monkey IgA (2500-fold diluted; Nordic-MUbio) in Block Ace was added and incubated at RT for 1 h. Wells were washed four times with PBS containing 0.05% (v/v) Triton X-100 between each step. Plates were developed at RT by adding 3,3′,5,5′-tetramethylbenzidine (Nacalai Tesque). The reaction was stopped with 1 M HCl and the absorbance was measured at 450 nm. Samples from mice or macaques obtained before immunizations were used as negative controls. The antibody titers were expressed as the reciprocal log2 of the highest dilution yielding an absorbance value that was 0.1 units greater than that of the negative control.

### Streptococcus pneumoniae

*S. pneumoniae* strain P1547 was obtained from Dr. Masamitsu Kono (Wakayama Medical University, Wakayama, Japan) [14], and *S. pneumoniae* strain ATCC 6303 was obtained from the American Type Culture Collection. A single *S. pneumoniae* colony was isolated from a Tryptic Soy (TS) broth plate or sheep blood agar plate and cultured at 37 °C with 5% CO_2_ overnight in the TS broth medium (BD).

### Pneumococcal infection in mice

One week after the mucosal boosting immunizations, *S. pneumoniae* strain P1547 (4.5 × 10^7^ colony-forming units) was used to intranasally challenge anesthetized mice. The mice were analyzed for survival rates.

### Pneumococcal infection in macaques

Two weeks after the mucosal boosting immunizations, *S. pneumoniae* strain ATCC 6303 (1 × 10^8^ colony-forming units) was used to intratracheally challenge anesthetized macaques. The macaques were analyzed for changes in body weight, histology, and blood tests.

Lung inflammation was investigated through thoracic computed tomography (CT) scanning on days 0, 2, and 6 after pneumococcal challenge. Thoracic CT was performed with the macaques under anesthesia (ketamine and xylazine) in a supine position. The CT images were obtained at 80 kV potential, 500 μA, and 160-mm field of view. An autopsy was performed on day 7 after the pneumococcal challenge.

Lung tissues from the indicated macaques 7 days after pneumococcal challenge were collected and washed with PBS. After fixation in 4% paraformaldehyde overnight, the tissues were embedded in paraffin. Five-micrometer sections were stained with hematoxylin and eosin and observed using a BZ-X800 microscope (Keyence).

Whole blood was collected into a blood collection tube and left to stand for at least 30 min at room temperature prior to centrifugation at 1,200 × *g* for 10 min. The C-reactive protein (CRP) levels of serum samples were determined at Kotobiken Medical Laboratories.

Numbers of bacteria in lung tissues were determined by counting colonies on sheep blood agar plates. Briefly, part of the lung tissue (from the same position in each macaque) was homogenized; the homogenized tissue was suspended in 1 mL of sterile PBS and 100 μL of the diluted sample was spread on a sheep blood agar plate. The plates were incubated overnight at 37 ℃ and 5% CO_2_.

### Statistical analyses

All statistical analyses were performed using EZR [15] (Saitama Medical Center, Jichi Medical University, Saitama, Japan), which is a graphical user interface for R (The R Foundation for Statistical Computing). More precisely, it is a modified version of R Commander designed to add statistical functions frequently used in biostatistics. Values of *P* < 0.05 were considered statistically significant. Error bars indicate the standard error of the mean (SEM).

## Results

### PspA3 + 2/WOW emulsion containing curdlan + CpG-ODN can induce antigen-specific serum IgG and lung IgA

We previously demonstrated that IFA emulsion of curdlan + CpG-ODN and antigen can strongly induce antigen-specific systemic immune responses as well as antigen-specific mucosal immune responses [1–3]. However, IFA is harmful to humans and its use as a vaccine adjuvant is prohibited. Therefore, we needed to substitute IFA with another agent.

Oil adjuvanted vaccines such as WOW have been used to enhance immune activity [16–18]. Because WOW emulsion vaccines have been used as human vaccine components, and are likely to produce fewer local reactions, we analyzed the effects of PspA3 + 2/WOW emulsion containing curdlan + CpG-ODN.

**Fig. 1:**
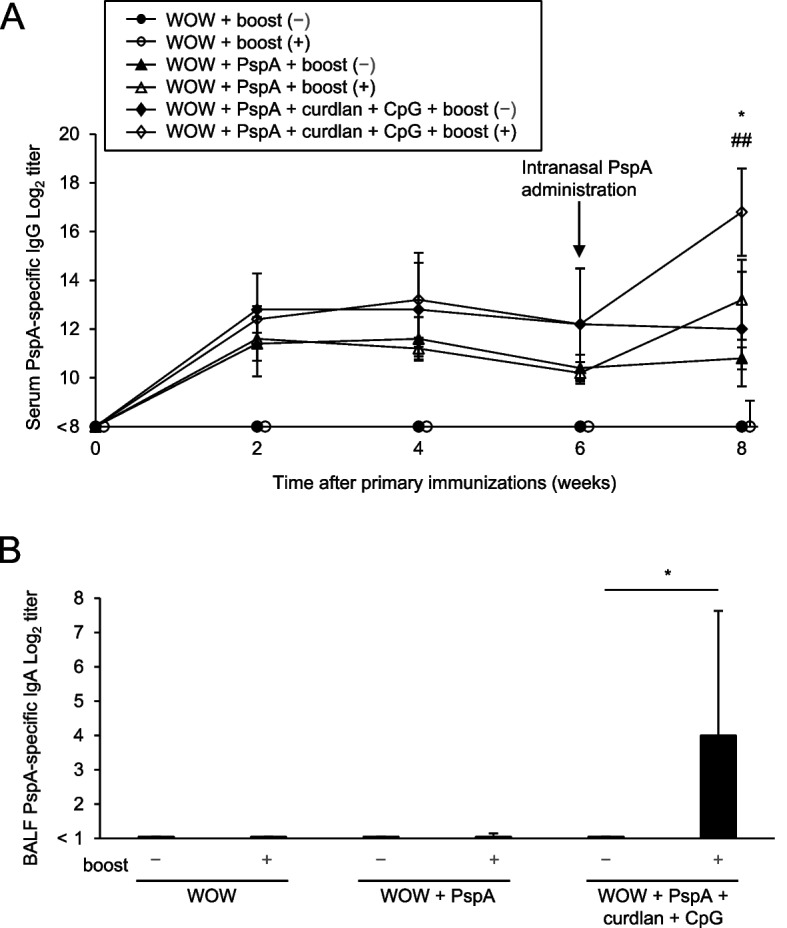
Nasal antigen administration boosts antigen-specific IgG in serum and IgA in BALF after primary immunization with PspA3 + 2/WOW emulsion containing curdlan + CpG-ODN. **A** Time course of PspA-specific serum IgG titers after primary immunization (*n* = 5 mice/group). Results represent mean ± SEM. *P < .05 (WOW + PspA vs. WOW + PspA + Boost),.^##^*P* < .01 (WOW + PspA + curdlan + CpG vs. WOW + PspA + curdlan + CpG + boost) (Tukey’s post hoc test). **B** Titers of PspA-specific IgA in bronchoalveolar lavage fluid (BALF) of mice immunized with PspA/WOW emulsion containing curdlan + CpG-ODN (*n* = 5 mice/group). Results represent mean ± SEM. *P < .05 (WOW + PspA + curdlan + CpG vs. WOW + PspA + curdlan + CpG + boost) (Tukey’s post hoc test)

Serum levels of PspA3 + 2-specific IgG increased after priming and remained steady thereafter by 6 weeks post-immunization (Fig. 1A). Six weeks after priming, we intranasally administered PspA3 + 2 alone. Levels of antigen-specific serum IgG were slightly increased 2 weeks after PspA3 + 2 mucosal boosting (Fig. 1A). In contrast, we induced PspA3 + 2-specific IgA production in the lungs by priming with curdlan + CpG-ODN and subsequent intranasal PspA3 + 2 administration (Fig. 1B). Therefore, as well as IFA-emulsified vaccines that we previously reported [1], WOW-emulsified PspA3 + 2 vaccine adjuvanted with curdlan + CpG-ODN induced antigen-specific serum IgG and lung SIgA.

### PspA3 + 2-boosting effect prevents S. pneumoniae infection

**Fig. 2:**
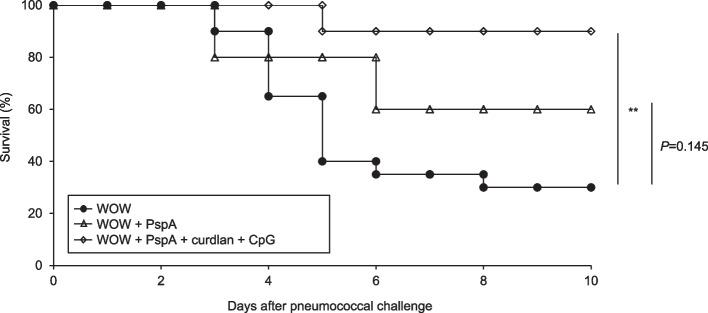
Vaccination with PspA3 + 2/WOW emulsion containing curdlan + CpG-ODN effectively controls pneumococcal infection. Kaplan-Meier survival analyses of mice immunized with PspA/WOW emulsion containing curdlan + CpG-ODN after challenge with *S. pneumoniae*. **P < .01 (vs. WOW) (log-rank Bonferroni post hoc test). WOW: *n* = 20 mice/group, other groups: *n* = 10 mice/group

We further examined the efficacy of PspA3 + 2/WOW immunization adjuvanted with curdlan + CpG-ODN in a mouse lung *S. pneumoniae* infection model. Six weeks after priming, PspA3 + 2 was administered intranasally and mice were then infected with *S. pneumoniae* strain P1547 (PspA family 1 clade 2, serotype 6A) 1 week later. Prime-boost-type PspA3 + 2/WOW immunization adjuvanted with curdlan + CpG-ODN significantly protected against fatal *S. pneumoniae* infection compared with WOW immunization and PspA3 + 2/WOW immunization (Fig. 2A). Thus, the induction of high titers of PspA3 + 2-specific IgA in the lung could be effective to prevent *S. pneumoniae* infection.

### Prime-boost-type vaccination of macaques effectively induces PspA3 + 2 immune responses

**Fig. 3:**
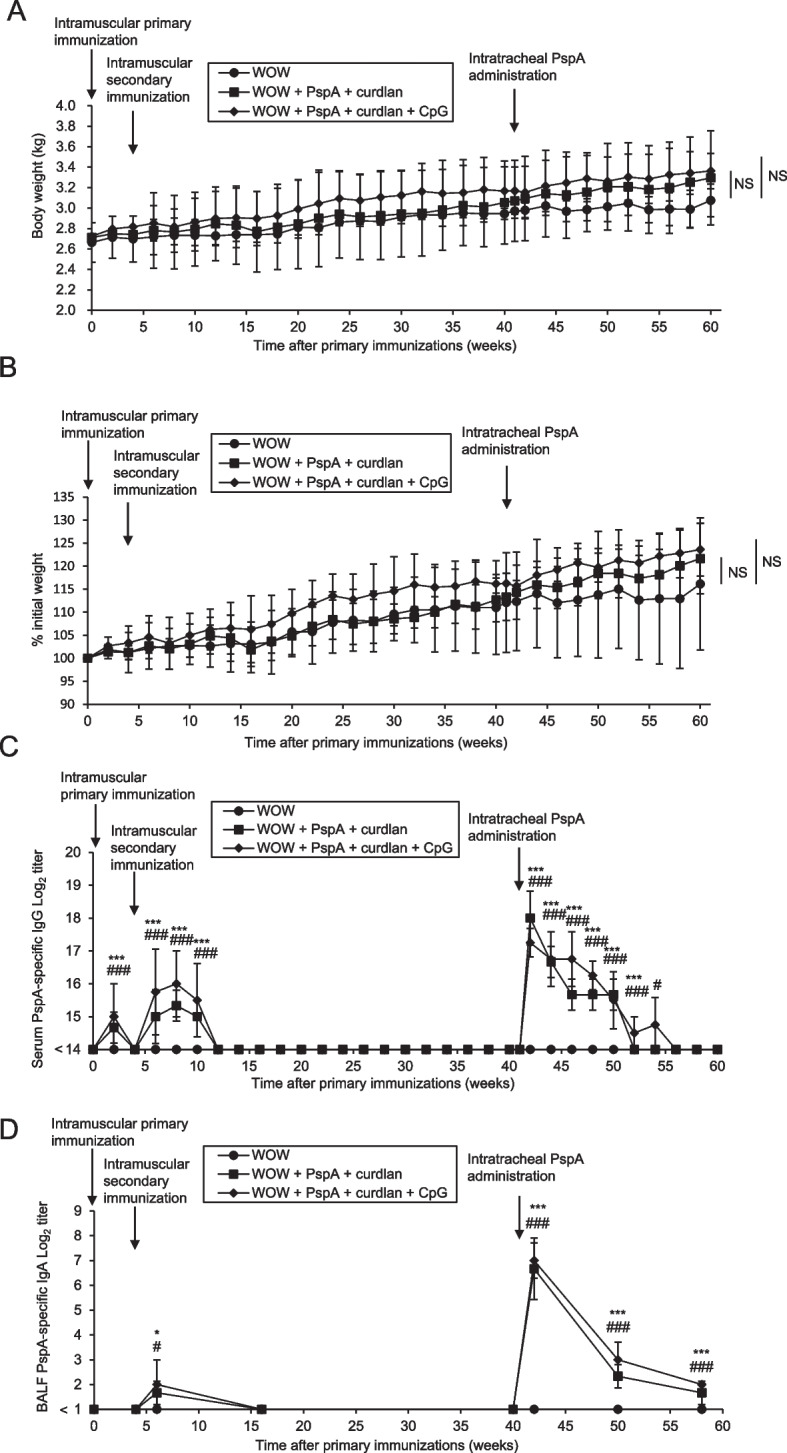
Prime-boost-type vaccination with curdlan and/or CpG-ODN can induce antigen-specific serum IgG and antigen-specific BALF IgA in macaques. **A**, **B** Body weight changes (**A**) and percentage body weight changes (**B**) after primary immunization. **C**, **D** Time course of PspA-specific IgG titers in the serum (**C**) and PspA-specific IgA titers in BALF (**D**) of macaques immunized with PspA/WOW emulsion containing curdlan and/or CpG-ODN. Results represent mean ± SEM. *P < .05 (naive vs. curdlan), ***P < .001 (naive vs. curdlan), ^#^*P* < .05 (naive vs. curdlan + CpG), ^###^*P* < .001 (naive vs. curdlan + CpG) (Tukey’s post hoc test). Naive and WOW + PspA + curdlan: *n* = 3 macaques/group, WOW + PspA + CpG + curdlan: *n* = 4 macaques/group

To obtain a proof-of-concept for the human application of prime-boost-type vaccine, 10 adult macaques were used to test the efficacy of the prime-boost vaccine (Table 1). Among them, three macaques were immunized with PspA3 + 2/WOW-emulsified curdlan, four macaques were immunized with PspA3 + 2/WOW-emulsified curdlan + CpG-ODN, and the remaining three macaques were injected with only WOW emulsion (Supplementary Figure S2A). No adverse effects on body weight change were observed at any time point in this study during the observation period (Fig. 3A, B). After the first immunization, curdlan and the combination of curdlan + CpG-ODN induced antigen-specific IgG in serum (Fig. 3C). However, antigen-specific SIgA in BALF was not detected by priming with curdlan alone or curdlan + CpG-ODN and subsequent intratracheal antigen administration (data not shown). Therefore, to induce antigen-specific SIgA in BALF effectively after intratracheal antigen boosting, additional intramuscular immunization was thought to be needed. Serum levels of antigen-specific IgG increased immediately after injection and peaked at 4 weeks after the second immunization (Fig. 3C). Furthermore, intratracheal PspA3 + 2 administration significantly increased antigen-specific IgG in serum (Fig. 3C). Unlike serum antigen-specific IgG, antigen-specific SIgA in BALF was not detected after the first immunization, but it was induced 2 weeks after the second injection (Fig. 3D). In addition, titers of antigen-specific SIgA in BALF were significantly increased after intratracheal PspA3 + 2 administration (Fig. 3D). These findings suggest that both antigen-specific serum IgG and antigen-specific BALF SIgA can be induced by prime-boost PspA3 + 2/WOW immunization adjuvanted with curdlan alone or curdlan + CpG-ODN.

**Table 1:** Characteristics of the individual macaques used in the study

Animal no	Age (years)	Sex	Body weight at start (kg)	Vaccination group	Serial no
1521707025	3	Female	2.75	WOW + PspA + curdlan + CpG	#001
1421707053	3	Female	3.01	WOW + PspA + curdlan + CpG	#002
1621707005	3	Female	2.7	WOW + PspA + curdlan + CpG	#003
1521706021	3	Female	2.73	WOW + PspA + curdlan	#004
1521709036	3	Female	3.05	WOW + PspA + curdlan	#005
1521709041	3	Female	2.46	WOW + PspA + curdlan	#006
1521711048	3	Female	2.45	WOW	#007
1621701001	3	Female	2.63	WOW	#008
1521708032	3	Female	3.01	WOW	#009
1421710097	3	Female	2.4	WOW + PspA + curdlan + CpG	#010
1421908086	3	Female	2.47	WOW + PspA + curdlan + CpG	#011
1521908055	3	Female	2.71	WOW	#012
1521907046	3	Female	2.15	WOW + PspA + curdlan	#013
1521912089	3	Female	2.51	WOW + PspA + curdlan + CpG	#014
1422002012	2	Female	2.4	WOW + PspA + curdlan	#016
1422002015	2	Female	2.23	WOW + PspA + curdlan + CpG	#017
1422004023	2	Female	2.25	WOW	#018
1421908078	3	Female	2.56	WOW + PspA + curdlan	#019
1421909101	3	Female	2.23	WOW + PspA + curdlan + CpG	#020

### Prime-boost-type mucosal vaccine protects macaques from pneumococcal infection

**Fig. 4:**
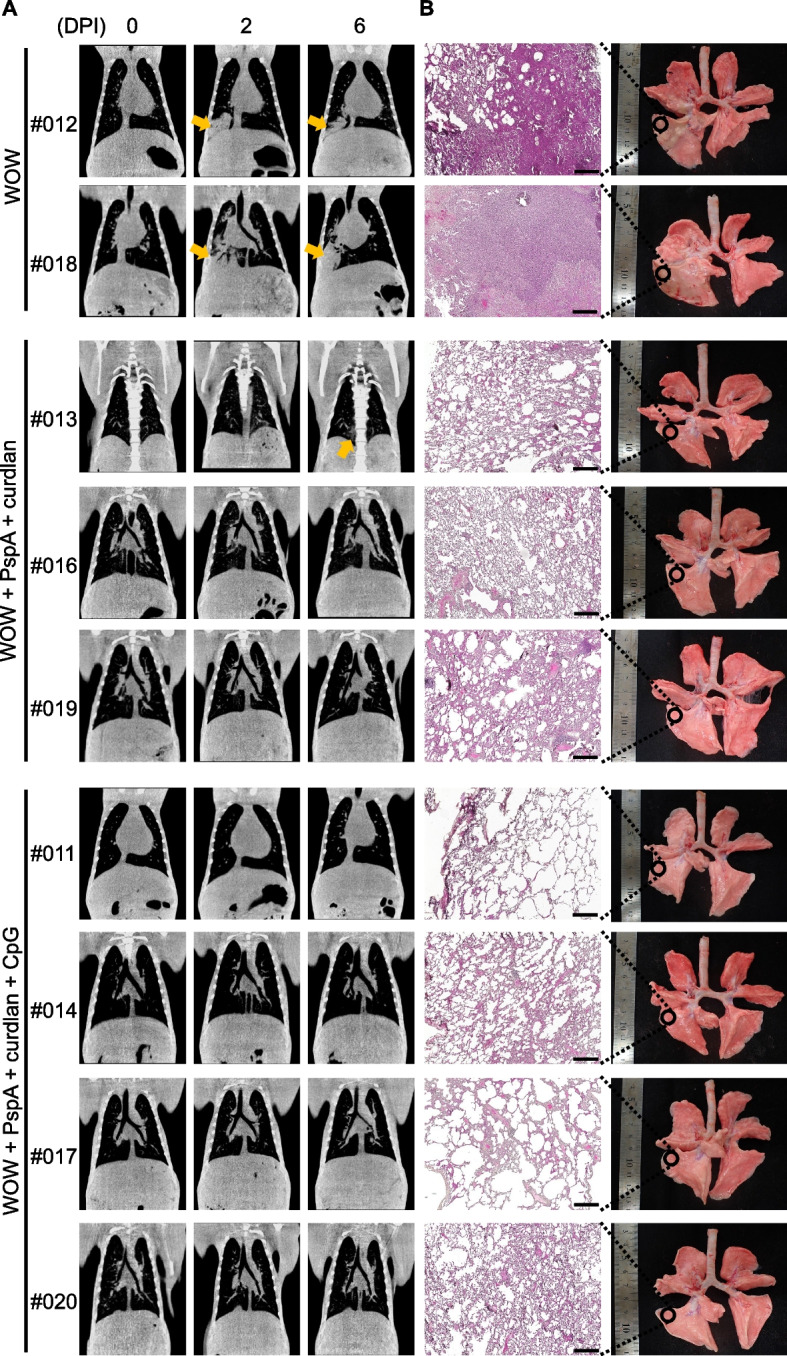
Thoracic computed tomography (CT) scans and histopathology of immunized macaque lungs after* S. pneumoniae* infection. **A** CT coronal plane images taken on the indicated days after *S. pneumoniae* infection are shown. DPI, day post-infection. Arrowheads indicate inflammation observed after *S. pneumoniae* infection. **B** Lung tissue in each macaque 7 days after *S. pneumoniae* infection was sectioned, stained with hematoxylin and eosin, and examined by microscopy. The scale bar represents 500 μm. Representative photos are shown

We further examined the protective effects of our vaccination using *S. pneumoniae*-induced pneumonia in macaques. Building on the previous investigation for which the results are shown in Fig. 3, three macaques were immunized with PspA3 + 2/WOW-emulsified curdlan, four macaques were immunized with PspA3 + 2/WOW-emulsified curdlan + CpG-ODN, and the remaining two macaques were injected with only WOW emulsion (Table 1). Thirteen weeks after the secondary intramuscular immunization, PspA3 + 2 was given intratracheally to all macaques (Supplementary Figure S2B). Two weeks later, the macaques were infected with *S. pneumoniae* strain ATCC 6303 (PspA family 2 clade 5, serotype 3), which is an IgA1 protease-positive *S. pneumoniae* virulent strain. A sublethal dose of *S. pneumoniae* strain ATCC 6303 was given intratracheally and lung inflammation was monitored by thoracic CT scan. In coronal plane views of the thorax, there were no indications of inflammation in any of the macaques before infection (Fig. 4A). WOW macaques #012 and #018 developed severe lung inflammation mainly in the posterior lobe of the right lung on day 2 after infection, and it was still present on day 6 after infection (Fig. 4A). Only WOW + PspA + curdlan macaque #013 developed slight pneumonia in part of the posterior lobe on day 6 after infection, but the other two immunized macaques #016 and #019 showed no clinical signs of lung inflammation (Fig. 4A). Macaques #011, #014, #017, and #019 from the WOW + PspA + curdlan + CpG immunization group also showed no clinical signs of lung inflammation (Fig. 4A). Furthermore, to analyze the pulmonary pathology, all macaques were euthanized 6 days after infection with pneumococcal strain ATCC6303 and their lungs were isolated. Gross findings of lung tissue showed extensive liver-like appearances and adhesions in the right lung in both of the two cases in the WOW group (Fig. 4B). Adhesion to the pleura was observed in macaque #19 from the WOW + PspA + curdlan group, but no obvious liver-like appearance was observed in macaque #19 from the WOW + PspA + curdlan group (Fig. 4B). Only one macaque #14 from the WOW + PspA + curdlan + CpG group showed a partial liver-like appearance (Fig. 4B). Examination of representative tissue sections revealed inflammatory-cell infiltration in the lung of WOW macaques #012 and #018, whereas the degree of infiltration was much smaller in the lungs of the other immunized macaques (Fig. 4B). These histopathological findings are consistent with the pneumonia observed by CT scans.

**Fig. 5:**
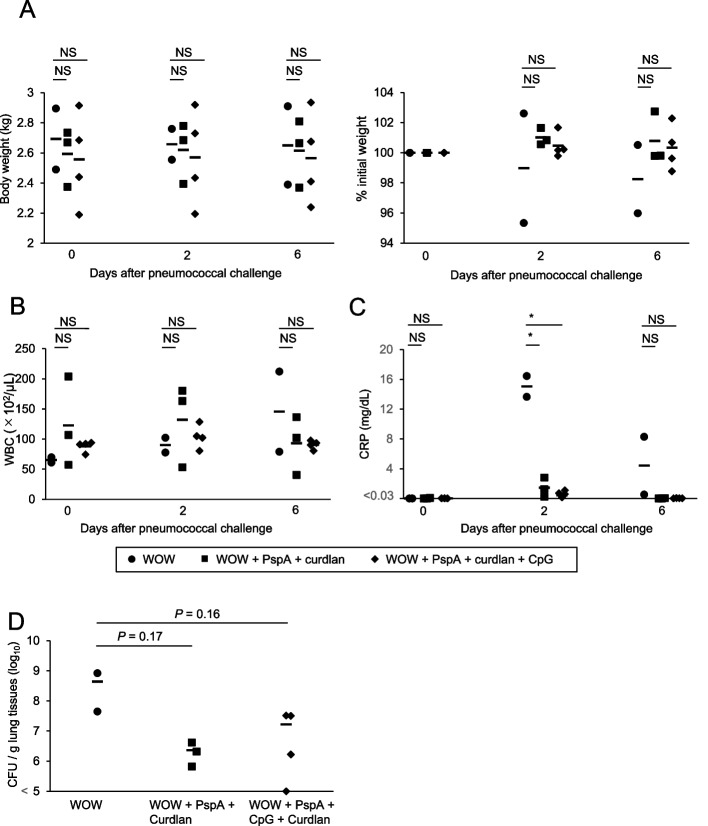
Time course of body weight, number of white blood cells, level of CRP, and number of bacteria in lung homogenates. **A** Body weight changes after *S. pneumoniae* infection are shown. **B** Numbers of white blood cells (WBCs) are shown. **C** Levels of C-reactive protein (CRP) are shown. **D** Numbers of bacteria in lung homogenates were evaluated by colony formation on sheep blood agar plates. CFU, colony-forming units. *P < .05 (vs. WOW). NS, not significant (Tukey’s post hoc test). WOW: *n* = 2 macaques/group, WOW + PspA + curdlan: *n* = 3 macaques/group, WOW + PspA + CpG + curdlan: *n* = 4 macaques/group

No significant changes in body weight were observed 2 and 6 days after infection with *S. pneumoniae* strain ATCC 6303 between the three groups (Fig. 5A). Whereas the number of white blood cells did not differ significantly among the three groups (Fig. 5B), the levels of C-reactive protein were significantly elevated in macaques #012 and #018 from the WOW group compared with the other two groups (Fig. 5C). In addition, examination of bacterial colonization of the lungs by counting colonies on blood agar plated with lung homogenates revealed increased pneumococcal numbers in the lungs of WOW macaques #012 and #018. The lung bacterial counts were much lower in macaques #013, #016, and #019 from the WOW + PspA + curdlan group and macaques #011, #014, #017, and #020 from the WOW + PspA + curdlan + CpG group (Fig. 5D). Thus, prime-boost-type immunization with PspA3 + 2/WOW-emulsified curdlan or PspA3 + 2/WOW-emulsified curdlan + CpG protected macaques from pneumococcal infection.

## Discussion

We substituted IFA-emulsified prime-boost mucosal vaccine with WOW-emulsified prime-boost mucosal vaccine and analyzed its protective effects against *S. pneumoniae* in mice and macaques.

In previous studies, we developed a new injectable vaccine that induces both systemic and mucosal immune responses using curdlan and CpG-ODN as adjuvants [1–3]. The curdlan + CpG-ODN immunized mice showed significantly reduced lung colonization by *S. pneumoniae* and less severe pneumonia [1]. The synergistic stimulation of conventional DCs by curdlan and CpG-ODN was essential for vaccine efficacy. Therefore, we used an IFA emulsion of curdlan + CpG-ODN and antigen at the immunization site. However, because the use of IFA as a vaccine adjuvant is prohibited in humans, the substitution of IFA with another agent will be necessary to enable the human application of our method [19–21]. In this study, PspA3 + 2/WOW immunization adjuvanted with curdlan + CpG-ODN induced antigen-specific serum IgG and BALF SIgA in mice (Fig. 1A, B). In addition, it was able to protect against *S. pneumoniae* infection (Fig. 2). These findings indicate that WOW-emulsified vaccine as well as the IFA-emulsified vaccine has the potential to induce antigen-specific mucosal immune responses to prevent pneumococcal infection.

Non-human primates are pivotal model systems for evaluating the safety and efficacy of pneumococcal vaccines [22], and have been recommended for the preclinical evaluation of new vaccine candidates [23]. The production of interleukin (IL)-6, retinoic acid (RA), and transforming growth factor (TGF)-β by dendritic cells (DCs) is critical for B cell IgA class-switch recombination [24, 25]. Although conventional DCs (cDCs) do not express RALDH2 and TGF-β, we previously demonstrated that curdlan induced TGF-β and CpG-ODN induced *Aldh1a2* in cDCs [1]. Interestingly, oil-based nanoemulsions such as oil-in-water itself have been shown to be able to induce RALDH [26]. In this study, antigen-specific lung SIgA could be induced by prime-boost PspA3 + 2/WOW immunization adjuvanted with curdlan alone or curdlan + CpG-ODN in macaques (Fig. 3D). Although the mechanism behind the induction of IgA might differ slightly between mice and macaques, it is thought that the WOW emulsion complements the function of CpG-ODN to induce *Aldh1a2*. Because PspA3 + 2/WOW immunization adjuvanted with curdlan could not induce antigen-specific BALF SIgA in mice (Supplementary Figure S3), it was difficult for us to predict whether lung IgA could be induced even in the absence of CpG-ODN in macaques. In humans, PspA3 + 2/WOW immunization adjuvanted with curdlan alone might be able to induce SIgA in mucosal tissues. Thus, identifying the optimal formulation for our innovative vaccine in human applications will be an interesting direction for future work.

Certain bacterial species produce proteases that play important roles in pathogenesis [27]. Among them, bacterial IgA1 proteases have been shown to interfere with the protective effects of IgA [28]. In 1979, IgA1 protease was identified in *S. pneumoniae* [29]. Therefore, *S. pneumoniae* IgA1 proteases were thought to have the potential to prevent the protective effects of IgA. In a previous study [1] and the current one, we demonstrated the efficacy of our vaccination against pneumococcal infection in mice (Fig. 2). However, mouse IgA is not cleaved by pneumococcal IgA1 protease [30], and these experiments did not reveal the virulence of IgA1 protease of *S. pneumoniae*.

In contrast to mouse IgA, IgA from macaques can be cleaved by pneumococcal IgA1 protease [31]. In this study, we used the IgA1 protease-positive *S. pneumoniae* strain ATCC 6303. We demonstrated that the curdlan only or curdlan + CpG-ODN immunized macaques showed less severe pneumoniae and ruduced lung colonization by *S. pneumoniae* after challenge (Figs. 4A, B and 5D), indicating the efficacy of PspA3 + 2/WOW immunization adjuvanted with curdlan or curdlan + CpG-ODN against pneumococcal infection (Fig. 4A, B). Given the human use of our prime-boost mucosal vaccine against pneumococci, the overcoming of the IgA protease virulence at the preclinical stage is remarkable and should lead to future clinical applications.

In summary, our prime-boost-type mucosal vaccines effectively inhibited pneumococcal infection. Compared with PCV13 and PPSV23, boosting respiratory mucosa, including nasal mucosa, rather than intramuscular injection, might be particularly beneficial for increasing the titers of antigen-specific IgG and IgA. Future clinical application of our vaccination strategy is strongly desired.

### Supplementary Information

**Additional file 1**: **Supplementary Figure S1.** Immunization schedule for mice. Six weeks after the primary immunizations, the mice received PspA boosters administered intranasally. **Supplementary Figure S2.** Immunization schedule for macaques. Macaques are immunized intramuscularly on week 0 and week 4. (A) Forty-one weeks after the primary immunizations, the macaques received PspA boosters administered intratracheally (related to Figure 3). (B) Seventeen weeks after the primary immunizations, the macaques received PspA boosters administered intratracheally (related to Figs. 4 and 5). **Supplementary Figure S3.** Nasal antigen administration do not boost antigenspecific IgA in BALF after primary immunization with PspA3+2/WOW emulsion containing curdlan in mice. Titers of PspA-specific IgA in bronchoalveolar lavage fluid (BALF) of mice immunized with PspA/WOW emulsion containing curdlan + CpG-ODN (*n*=5 mice/group). NS; not significant (Tukey’s post hoc test).

## Data Availability

All data generated or analyzed during this study are included in this published article.

## Abbreviations

PspA: Pneumococcal surface protein A
CpG-ODN: CpG oligodeoxynucleotide
IFA: Incomplete Freund’s adjuvant
WOW: Water-in-oil-in-water
NIBIOHN: National Institute of Biomedical Innovation Health and Nutrition
IPTG: Isopropyl β-D-thiogalactoside
PBS: Phosphate-buffered saline
ELISA: Enzyme-linked immunosorbent assay
BALF: Bronchoalveolar lavage fluid
RT: Room temperature
TS: Tryptic soy
CT: Computed tomography
CRP: C-reactive protein
SEM: Standard error of the mean
IL: Interleukin
RA: Retinoic acid
TGF: Transforming growth factor
DCs: Dendritic cells
cDCs: Conventional DCs
