# Supplementary material for: Prime-boost-type PspA3 + 2 mucosal vaccine protects cynomolgus macaques from intratracheal challenge with pneumococci
Source: Inflamm Regen. 2023 Nov 15;43:55. doi: 10.1186/s41232-023-00305-2 (PMC10647109; doi:10.1186/s41232-023-00305-2)
Supplement: Supplementary file 1 — Additional file 1: Supplementary Figure S1. Immunization schedule for mice. Six weeks after the primary immunizations, the mice received PspA boosters administered intranasally. Supplementary Figure S2. Immunization schedule for macaques. Macaques are immunized intramuscularly on week 0 and week 4. (A) Forty-one weeks after the primary immunizations, the macaques received PspA boosters administered intratracheally (related to Figure 3). (B) Seventeen weeks after the primary immunizations, the macaques received PspA boosters administered intratracheally (related to Figs. 4 and 5). Supplementary Figure S3. Nasal antigen administration do not boost antigenspecific IgA in BALF after primary immunization with PspA3+2/WOW emulsion containing curdlan in mice. Titers of PspA-specific IgA in bronchoalveolar lavage fluid (BALF) of mice immunized with PspA/WOW emulsion containing curdlan + CpG-ODN (n=5 mice/group). NS; not significant (Tukey’s post hoc test). [file 41232_2023_305_MOESM1_ESM.pdf]

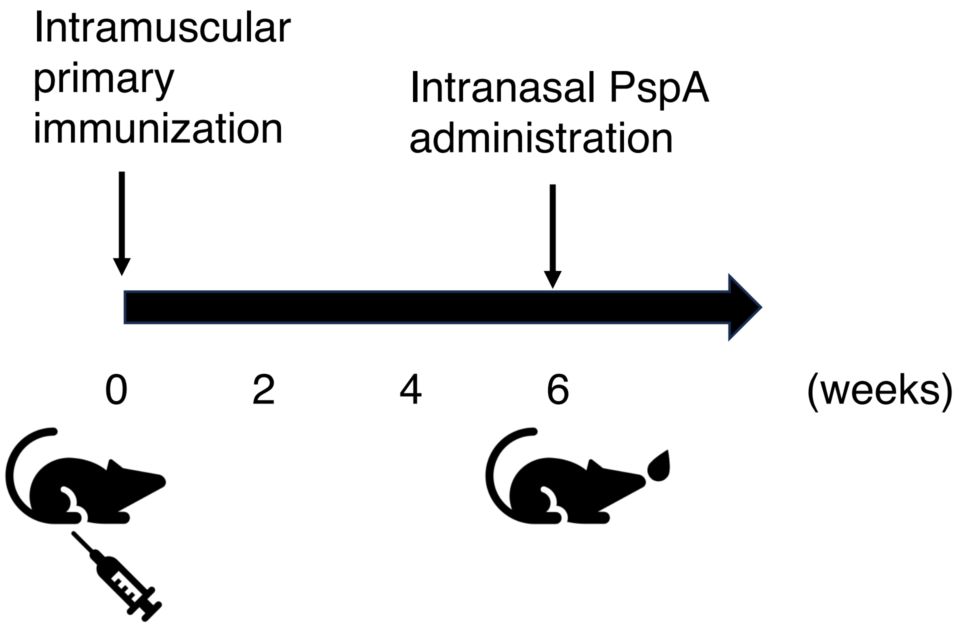

Supplementary Figure 1. Immunization schedule for mice.

Six weeks after the primary immunizations, the mice received PspA boosters administered intranasally.

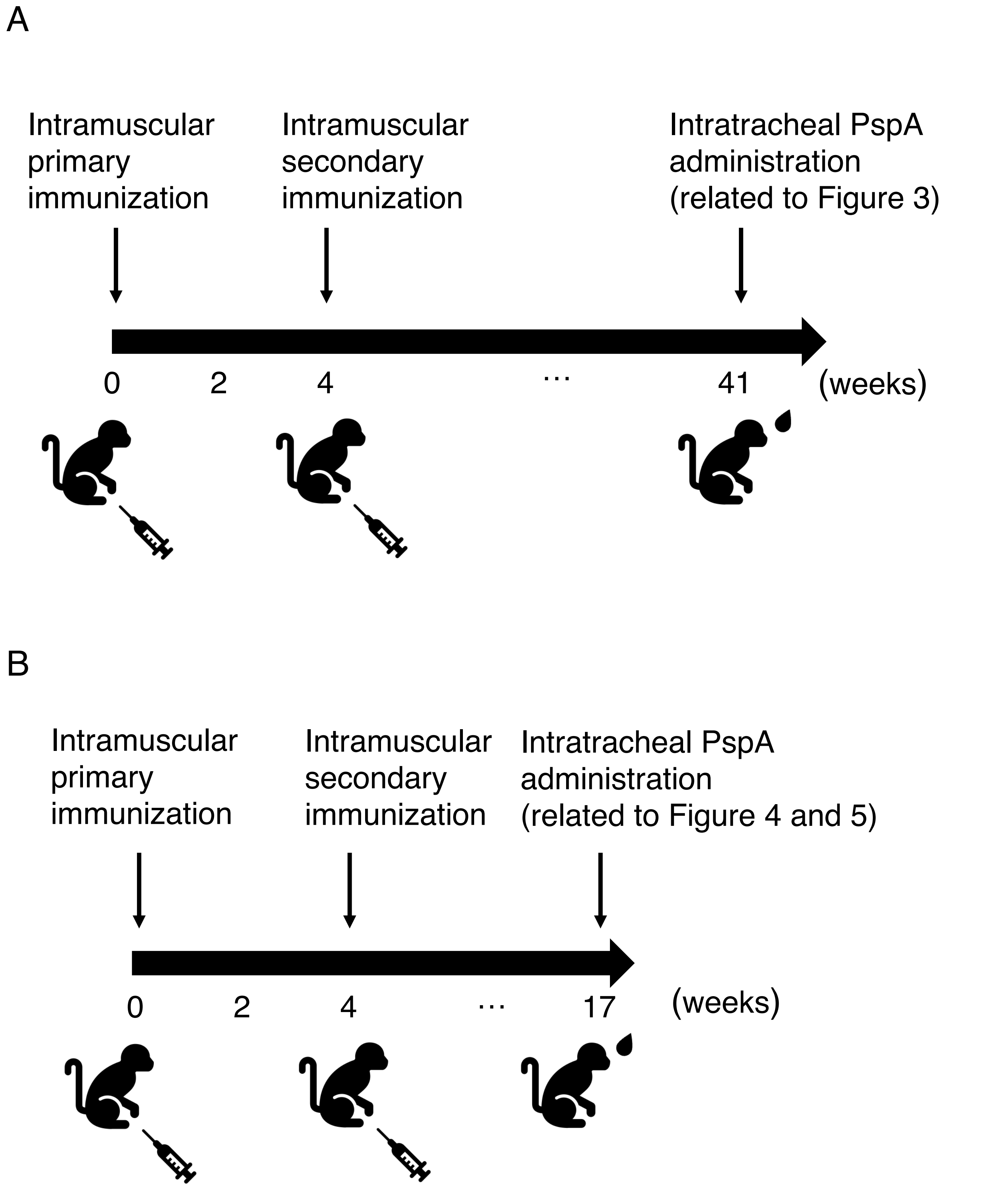

Supplementary Figure 2. Immunization schedule for macaques.

Macaques are immunized intramuscularly on week 0 and week 4.

(A) Forty-one weeks after the primary immunizations, the macaques received PspA boosters administered intratracheally (related to Figure 3).

(B) Seventeen weeks after the primary immunizations, the macaques received PspA boosters administered intratracheally (related to Figure 4 and 5).

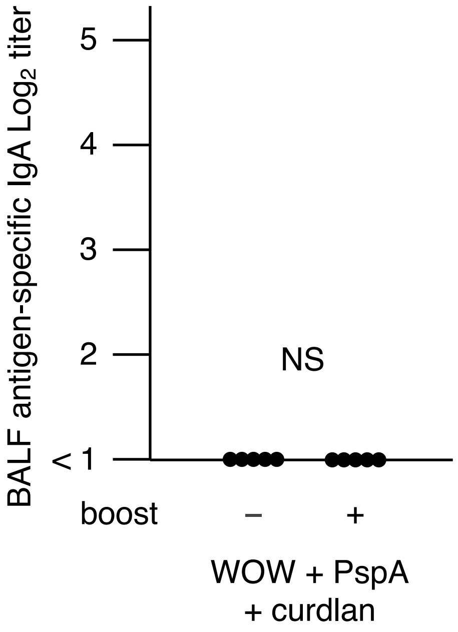

Supplementary Figure 3. Nasal antigen administration do not boost antigen-specific IgA in BALF after primary immunization with PspA3+2/WOW emulsion containing curdlan in mice.

Titers of PspA-specific IgA in bronchoalveolar lavage fluid (BALF) of mice immunized with PspA/WOW emulsion containing curdlan + CpG-ODN (n=5 mice/group). NS; not significant (Tukey's post hoc test).
